# Effects of mild psychological stress on facial impressions

**DOI:** 10.3389/fpsyg.2023.1186046

**Published:** 2023-06-29

**Authors:** Koyo Koizumi, Naoyasu Hirao, Haruna Yamanami, Hideki Ohira

**Affiliations:** ^1^Brand Value R&D Institute, Shiseido Co., Ltd., Yokohama, Japan; ^2^MIRAI Technology Institute, Shiseido Co., Ltd., Yokohama, Japan; ^3^Department of Psychology, Graduate School of Informatics, Nagoya University, Nagoya, Japan

**Keywords:** facial impression, psychological stress, blood flow, survey, Stroop Color-Word Test

## Abstract

**Introduction:**

Appearance plays an important role in maintaining a positive impression in social interactions. Psychological stress is known to have an adverse effect on facial skin, as indicated in previous studies. However, no study has investigated the negative effect of stress on facial impressions. Therefore, we aimed to investigate changes in impressions from facial images before and after mental stress tasks using an online survey.

**Method:**

Thirteen Japanese men were recruited to have their facial photographs taken before and after undergoing a psychological stress task. We observed the physiological effects of an increased heart rate and decreased blood flow on the cheek skin. Four average facial images were created for each time point (control: “baseline;” stress: “0H,” “1H,” and “3H”) from their facial photographs. An online survey was conducted with 700 Japanese participants, who compared the “baseline” to other images and selected one of two options in each of the six questionnaire items of impressions.

**Results:**

The results showed that the rate of participants who chose “baseline” was significantly lower in the items “looks tired,” “looks old,” and “looks irritated” and higher in “looks clean-cut” and “looks healthy” compared to other images created from photographs after the stress task (“0H,” “1H,” and “3H”).

**Conclusion:**

These results suggest that psychological stress loading not only causes physiological changes in autonomic nervous activity and skin blood flow but also negatively impacts facial impressions for a few hours following a mild stress load.

## 1. Introduction

In social situations, individuals often prioritize maintaining a positive facial impression, as negative impressions can be detrimental to interpersonal communication. Consequently, individuals use cosmetics such as skincare and makeup to improve facial impressions. Skincare can keep skin healthy by improving skin condition, whereas makeup can correct skin brightness and color. Cosmetics are believed to be effective in making a favorable impression on others. Numerous previous studies have investigated the relationship between facial impressions and features such as facial surface topography, color, and radiance (Fink et al., 2006, 2018; Ikeda et al., 2021). Furthermore, research has also been conducted on the effects of wearing masks during the recent COVID-19 pandemic on socio-cognitive processes, suggesting that facial appearance affects interpersonal communication (Castelli et al., 2022; Villani et al., 2022).

In modern times, many individuals experience mental stress on a daily basis, which is known to adversely affect their skin condition and cause changes in facial features (Chen and Lyga, 2014). The autonomic nervous system plays a significant role in regulating the effects of stress, with the sympathetic nervous system becoming dominant under stress leading to an increase in heart rate (Skoluda et al., 2015). Furthermore, stress affects blood flow regulation through the autonomic nervous system, resulting in changes in facial blood flow due to sympathetic vasoconstriction and vasodilation. For example, Sokhadze (2007) reported that presenting a visual stimulus that induces disgust for 20 s can cause a reduction in forehead blood flow. Furthermore, a calculation task can induce blood flow in the earlobe (Watanabe and Kageyama, 2017). This study also reported that facial skin color can change due to decreased blood flow. These changes in skin appearance, partly due to changes in blood flow, might also affect facial impressions. However, to date, no study has investigated the negative effect on facial impressions, which may have a negative influence on social communication due to the influence of psychological stress on the skin.

Therefore, in this study, we focused on stress as a factor that may affect short-term impression changes in daily life. Deterioration of facial impressions due to psychological stress can negatively impact interpersonal communication. Therefore, understanding the impact of stress is crucial not only for our health but also for social interactions and can help us in taking precautions to avoid potential harm in our daily communications. Additionally, knowing how facial impressions change in response to stress can help in developing more effective cosmetic products that can both care for and conceal the negative effects of stress on the skin.

In this study, we investigated whether short-term, mild stress that can occur in daily life changes facial impressions. First, a psychological stress load test was conducted to acquire facial images before and after a psychological stress load. The Stroop Color-Word Test was used as a mild stress load, similar to a previous study by Skoluda et al. (2015). Heart rate and facial blood flow were measured to confirm whether changes in the autonomic nervous system activate due to stress load comparisons. An online impression survey was conducted using average facial images created from facial images taken before and after stress loading. In this study, we utilized the online survey method, which was also used in our previous study that investigated the differences in impressions associated with skin color (Hirao et al., 2021). Evaluation of facial images was conducted in paired comparisons in each of six impression words, and facial images before stress were compared with three facial images after stress (0, 1, and 3 h after stress load). Decreased blood flow due to stress can cause changes in skin color (Watanabe and Kageyama, 2017). In our hypothesis, the change in skin color’s brightness is related to the impression of “tired” and “old,” and the change in redness affects the impression of “irritated.” The changes in impressions of facial images after stress loading in these impression questions could provide evidence that stress affects facial impressions. These results suggest the negative influence of mental stress extending on social communication and are one of the first steps to developing skincare and makeup products to improve the impression of individuals in a stressful society.

## 2. Materials and methods

### 2.1. Creation of averaged facial images before and after psychological stress load task

#### 2.1.1. Volunteers

A total of 13 healthy Japanese men (aged 29–42) volunteered to create averaged facial images before and after a psychological stress load task for the impression survey. The mean age of the volunteers was 34.2 years (SD = 4.2). Each volunteer provided informed consent. The Research Ethics Committee of the Shiseido Global Innovation Center approved this study, and all methods were followed according to the approved guidelines.

#### 2.1.2. Procedure

**Figure 1:**
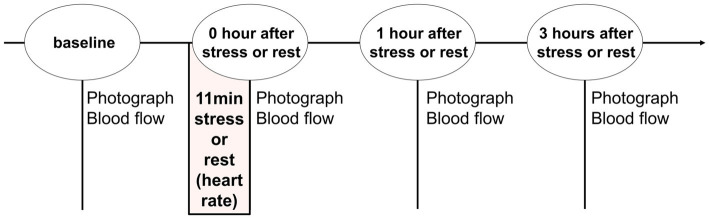
Study design and procedure. Facial photography and blood flow measurements were recorded at baseline and 0, 1, and 3 h after stress or rest. Stress or rest was set between 45 and 60 min after entering the laboratory.

We recruited volunteers to acquire facial images before and after psychological stress. Furthermore, to confirm the occurrence of stress load, data were also obtained when there was no stress load as a control. Therefore, the volunteers participated on 2 days, one stress day (stress condition) and one stress-free day (rest condition). The order of stress and rest was randomized for each volunteer. The experimental procedure is illustrated in Figure 1. In the laboratory, air temperature and humidity were controlled at 23 degrees centigrade and 45%, respectively, photographs of their faces were taken, and blood flow of facial skin was measured before stress or rest (baseline) and 0, 1, and 3 h after stress or rest in each condition. In addition, volunteers waited in a sitting position in the laboratory, except during measurement.

#### 2.1.3. Psychological stress load task

**Figure 2:**
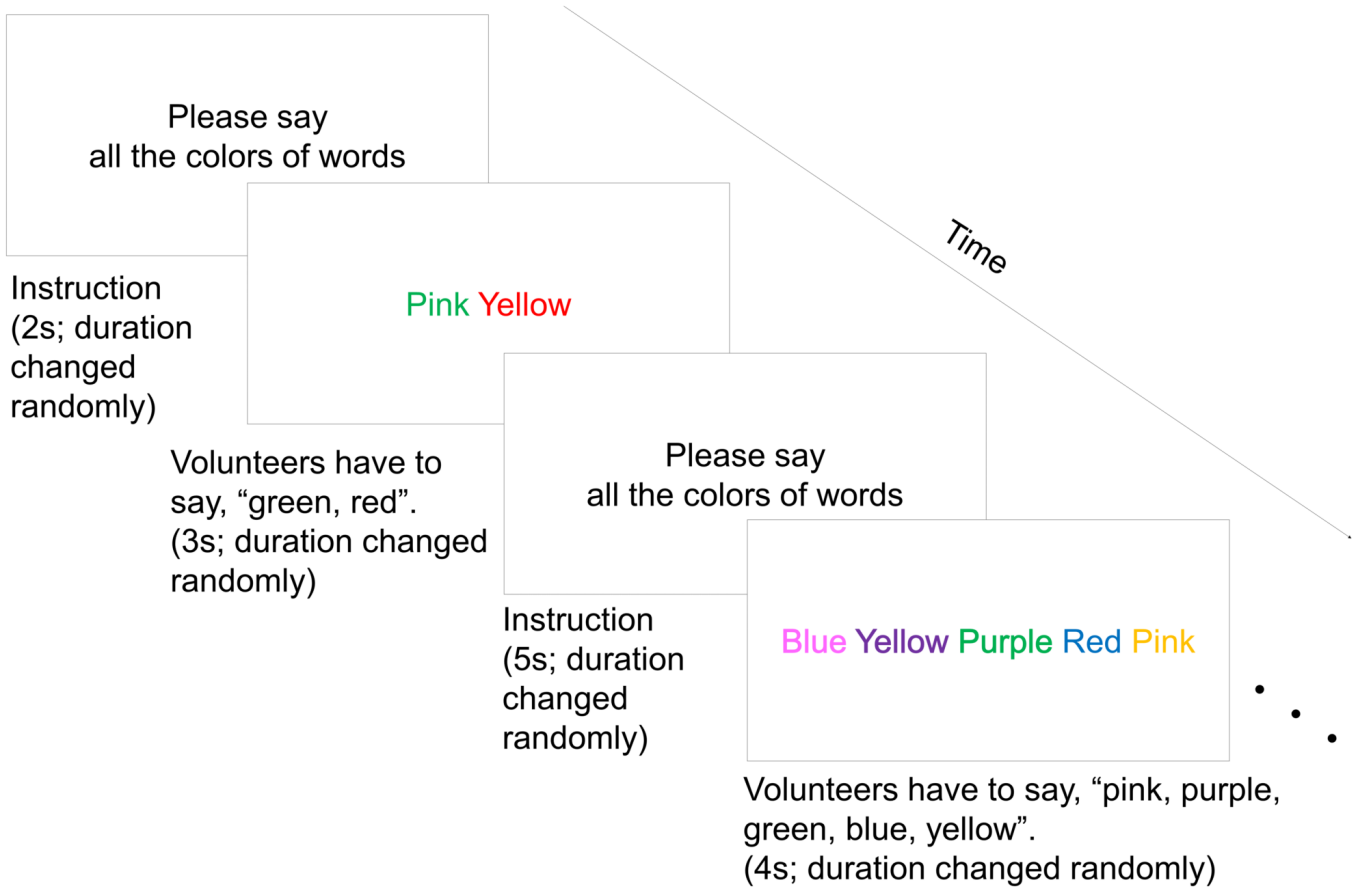
An image of the Stroop Color-Word Test. Colored words were shown simultaneously, and volunteers had to say all the colors of words on the screen before changing the slide. A maximum of seven color words were displayed on the monitor. The seven color words were blue, yellow, green, pink, purple, red, and light blue. The number of color words displayed on one slide randomly changed from 1–7. The slide was displayed automatically and switched to the next slide at random intervals of 1–8 s. The interval between stimuli was randomly 1–6 s by inserting instruction slides between word slides. A total of 100 slides were presented as stimuli.

As a short-term mild psychological stress load task in the stress condition, we conducted an improved Stroop Color-Word Test referencing Skoluda et al. (2015) method. The volunteers were instructed to respond verbally to the color names of letters presented on a monitor. A maximum of 7 color words were displayed on the monitor: blue, yellow, green, pink, purple, red, and light blue. The number of color words displayed on one slide randomly changed from 1–7. The slide was displayed automatically and switched to the next slide at random intervals of 1–8 s. The interval between stimuli was randomly 1–6 s by inserting instruction slides between word slides. The number of color words presented, display duration, and interval duration were randomized for the purpose of increasing stress. A total of 100 slides were presented as stimuli. The duration was 11 min. Figure 2 shows an image of the Stroop Color-Word Test. The Stroop effect showed that the reaction was delayed when the participants tried to verbally respond to the color of a word whose color did not match the character. This represented the color name compared to simply responding to the color of the character (Stroop, 1935). In this study, the Stroop Color-Word Test was administered to provide a psychological stress load. In addition, to increase stress, the participants were instructed to respond to the color names of the letters as soon as possible. Furthermore, if participants responded incorrectly or took too long to respond, an error sound was played. We retained a record of the number of incorrect responses by counting the number of times volunteers responded incorrectly or did not respond in time. In contrast, volunteers remained still for 11 min in the rest condition. A researcher explained and monitored the test, played error sounds, and remained in the same room throughout the test.

#### 2.1.4. Heart rate

When psychological stress is experienced, and the sympathetic nervous system becomes dominant, the heart rate increases (Skoluda et al., 2015). We measured heart rate, with a wearable heart rate sensor (myBeat WHS-1; Union Tool Co., Tokyo, Japan), as an index of the psychological stress load. It calculated the heart rate every 30 s through an electrocardiogram, measured by placing two electrodes on the left side of the chest. The participants’ heart rates were measured during the Stroop test or rest condition (11 min) and when relaxed (2 min) before and after the two conditions. The participants were seated at rest for 2 minutes before and after the stress or rest conditions.

#### 2.1.5. Blood flow on facial skin

Facial blood flow was also measured at baseline and 0, 1, and 3 h after stress or rest. Blood flow was measured on the cheek and under the eye via a two-dimensional laser blood flow meter (OMEGAZONE OZ-2; OMEGAWAVE, INC., Tokyo, Japan). The meter signaled the scattered light from red blood cells and detected the red blood cell count and velocity. The resulting output was a value that corresponded to the blood volume “mL/min/100 g” that flowed into the unit tissue weight.

#### 2.1.6. Statistical analysis of physiological response

Paired *t*-tests were conducted to assess the significant differences between stress and rest condition in heart rate and blood flow. The *p*-values were corrected by Bonferroni’s method. The significance level *α* was set at *α* = 0.05.

#### 2.1.7. Face image

Photographs of the face were taken at baseline and at 0, 1, and 3 h after stress or rest before the measurement of blood flow to create stimuli for the impression survey. The face photographs were taken from the front. The camera was positioned to ensure that the head and chest were both visible in the image. Volunteers were requested to remain seated and expressionless and keep their eyes open and mouths closed for the photographs. Shutter speed and diaphragm of the camera (EOS-1Ds Mark III; Canon Inc., Tokyo, Japan) were set at 1/125 s and f/22, respectively.

#### 2.1.8. Stimuli for impression survey

**Figure 3:**
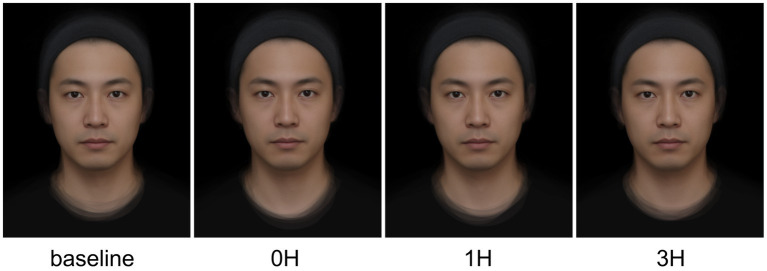
Average face images. The process of creating an average face involved capturing facial images from 13 individuals before and after exposure to a stressful condition. The images were obtained at different time points: baseline (control), 0, 1, and 3 h after exposure to stress. These were subsequently analyzed using image analysis software installed on a personal computer. The facial landmarks were identified, and the shape of the face image was averaged based on the marker point coordinates. The colors and brightness were also averaged for the colors at the same point. The resulting image was the average face, which represented the group’s overall facial features before and after exposure to stress.

Average facial images were created by averaging the images of 13 volunteers taken at baseline, 0, 1, and 3 h after stress in the stress condition. One facial image was prepared for each volunteer, and a total of 13 facial images were used to create an average face. Average faces were created for four patterns at baseline and 0, 1, and 3 h after the stress load. The shape of the face image was averaged based on the marker point coordinates of the acquired face image with the image analysis software (Abrosoft FantaMorph Deluxe) installed on a personal computer (manufactured by Panasonic). The colors and brightness were averaged for the colors at the same point. Four average facial images (control: “baseline;” after stress: “0H,” “1H,” and “3H”) were obtained. The size of these images was 300 × 400 pixels. The images created are shown in Figure 3.

### 2.2. Impression survey

#### 2.2.1. Participants

A total of 700 healthy individuals who lived in Japan (350 men and 350 women; mean age: 43.5 years; SD: 10.0) participated in the survey. The number was considered sufficient to detect a 10% difference in selection rate in the chi-square test in the condition of *α* = 0.05 and 1−*β* = 0.8. None of the participants indicated optical disorders via a self-report before the experiment, and everyone owned a personal computer or tablet, which excluded mobile phones with a small display. Each participant provided informed consent. The Research Ethics Committee of the Shiseido Global Innovation Center approved this study, and all the methods were followed according to the approved guidelines.

#### 2.2.2. Procedure

The relationship between facial features and impressions has been studied in experiments using facial images (Samson et al., 2010a; Jaeger et al., 2018). These studies are generally conducted in the laboratory where facial images are displayed on color- and brightness-controlled printed media or computer displays and evaluated under well-controlled lighting conditions. Unifying the visual conditions of the stimuli can reduce noise in the evaluation. However, online surveys are also used to evaluate facial images, and their advantage is that a large number of participants can be recruited from a wide range of regions at a relatively low cost per person. Additionally, reducing the risk of infections in a laboratory is also one of the major benefits. While the lack of control over monitors and lighting conditions used by the participants can increase the error in evaluating each stimulus, statistical errors can be reduced by increasing the number of participants. We compared results from a previous well-controlled laboratory experiment and an online survey (Hirao et al., 2021) and found that evaluation noise could be effectively offset by preparing a sufficient sample size compared to lab-based experiments. The online survey method allows us to investigate the effects on facial impressions caused by changes in facial features, resulting from various factors such as changes in skin condition due to the environment, physical, and mental factors.

**Figure 4:**
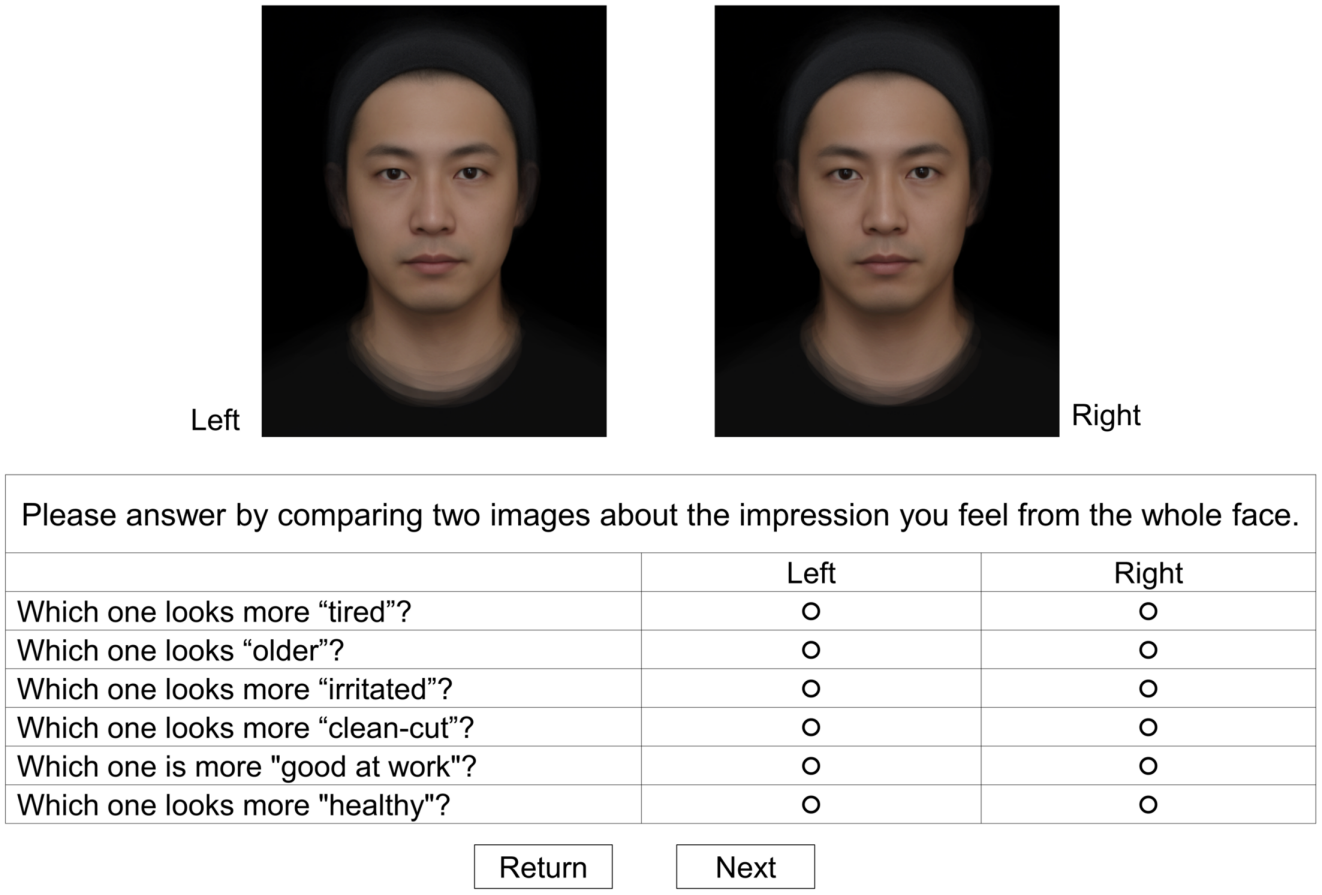
An image of the survey screen. Two facial images were displayed on the left and right sides of the monitor, and six questions were presented below the facial images. Participants compared the two face images and chose by clicking which face image, the left or right, was more suitable for the item. The display position and appearance order of the images were randomized. The order of the questions was also randomized for each participant and presented in the same order when the presented images were switched.

The survey was conducted by Marketing Center Co., Ltd. using a platform named Market Observer (GMO Research Co., Ltd.). It is a system in which responses can be made on a browser, regardless of the type. The participants looked at the stimuli on their personal computers or tablets and completed the questionnaire at home. At the beginning of the questionnaire, the participants were requested to select the terminal that they were using to respond to the questionnaire. Participants who chose anything other than a desktop, laptop, or tablet were excluded from the survey. Facial images were evaluated in paired comparisons. Two facial images were displayed on the left and right sides of the monitor, and six questions were presented below the facial images. We set six items related to the facial impression (“looks tired,” “looks old,” “looks irritated,” “looks clean-cut,” “looks good at work,” and “looks healthy”). Decreased blood flow due to stress can cause changes in skin color (Watanabe and Kageyama, 2017). In our hypothesis, we expected the change in skin color’s brightness to be related to the impression of “tired” and “old” and change in redness to be related to the impression of “irritated.” It is known that the brightness under the eyes affects the appearance of “health” (Jones et al., 2016). Dark circles have also been reported to be a sign of fatigue (Sundelin et al., 2013). Based on these findings, it was perceived that darker skin might provide the impression of being tired. Redness of the skin is also known to result in an impression of “aggression” (Stephen et al., 2012). Therefore, we adopted “irritated” as an impression word related to “aggression.” Furthermore, it has been reported that the age of a man can be predicted by skin color (Fink et al., 2012). Hence, we selected the term “old.” In addition to these negative questions, we added the same number of positive questions (“looks clean-cut,” “looks good at work,” and “looks healthy”). Positive questions were used to ensure that participants had carefully reviewed and responded. We assumed that if participants all chose the left or right image, there would be discrepancies in the selection rates of the positive and negative questions. Participants compared the two facial images and responded with which facial image was more suitable for the items. They repeated this for three pattern combinations (“baseline” vs. “0H,” “baseline” vs. “1H,” and “baseline” vs. “3H”). The images and questions were switched at the participant’s own pace, and they responded to each combination pattern once. The display position and appearance order of the images were randomized. The order of questions was also randomized for each participant and presented in the same order when the presented images were switched. Figure 4 shows an image of the survey screen.

#### 2.2.3. Statistical analysis

A pair of facial images were presented for each question. Participants were requested to respond with the image that they considered more appropriate for the question. The ratio of the number of participants who selected face images for each question was calculated (selection rate), and a chi-square test was used to determine if there was a significant difference in the face selection rate between conditions. The null hypothesis was that there would be no difference in the face image selection rate under any condition (expected values were 50% baseline and 50% after stress). The significance level *α* was set at *α* = 0.05.

## 3. Results

### 3.1. Heart rate & blood flow

**Figure 5:**
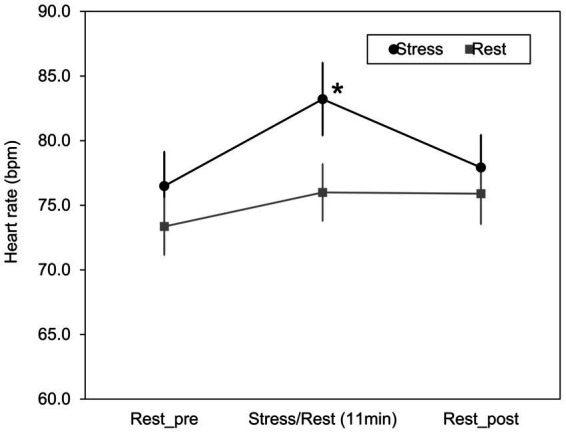
Changes in heart rate due to stress/rest condition. Changes in heart rate were measured with a wearable heart rate sensor as an index of psychological stress load during the Stroop test or rest (11 min) and 2 min before and after these conditions. Mean ± S.E., *n* = 13. *Significantly different from the value of stress/rest (*Bonferroni-corrected p* < 0.05).

A psychological stress load task was performed to acquire facial images after stress loading. In addition, by comparing heart rate and blood flow under the stress and rest conditions, it was confirmed whether psychological stress load occurred. Figure 5 shows the results of the heart rate for 11 min during the Stroop test (stress condition) or at rest (rest condition) and 2 minutes before and after these conditions. Data were acquired for 2 minutes before and after the stress and rest conditions. No significant difference in heart rate was observed in the relaxed state for 2 minutes before and after stress treatment and rest treatment. However, the heart rate during the 11 min period during the Stroop Color-Word Test increased significantly compared to that of the rest treatment [t(12) = 3.04, *Bonferroni-corrected p* = 0.031].

**Figure 6:**
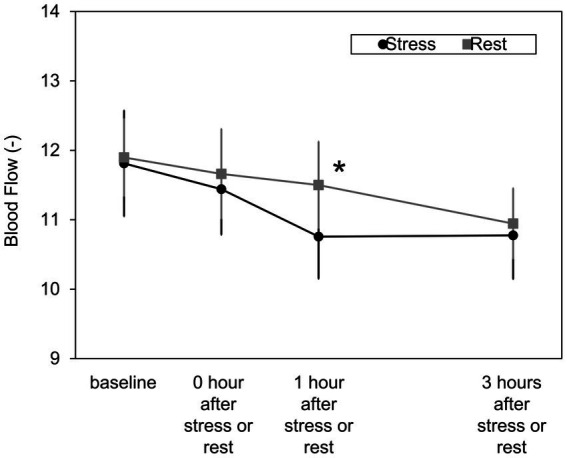
Changes in blood flow under the stress/rest condition. Blood flow on the cheek was measured using a two-dimensional laser blood flow meter. Mean ± S.E., *n* = 13. *Significantly different from the value of stress/rest (*Bonferroni-corrected p* < 0.05).

Figure 6 shows changes in cheek blood flow. Under the stress condition, a significant decrease in blood flow of the cheek was observed 1 hour after stress compared to the rest condition [t(12) = 4.28, *Bonferroni-corrected p* = 0.004].

### 3.2. Impression survey

**Figure 7:**
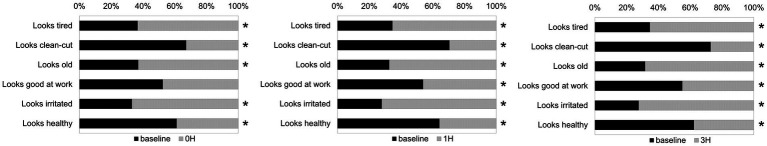
The face image selection rate for each impression question. In the impression survey, a set of paired facial images was presented for each question, and the participants were requested to select which image they thought was more appropriate for the given question. The ratio of the number of individuals who chose face images for each question was calculated. *Significantly different in the selection rate (chi-square test, *p* < 0.05).

Figure 7 shows the face image selection rate for each question. In order to investigate impression changes due to stress load, selection rates before (“baseline”) and after stress load (“0H,” “1H,” and “3H”) were calculated and compared. The skin color was thought to have changed because the blood flow decreased due to the stress load. Moreover, we thought that the change in skin color’s brightness would affect the impression of “tired” and “old,” and the change in redness would affect the impression of “irritated.” Positive questions were used to ensure that participants had carefully reviewed and responded. We assumed that if all participants chose the left or right image, there would be discrepancies in the selection rates of the positive and negative questions. Comparing the selection rates of “baseline” and after stress load (“0H,” “1H,” and “3H”) in negative impression questions (“looks tired,” “looks old,” and “looks irritated”), the selection rate of “0H,” “1H,” and “3H” was significantly higher than “baseline” in all impression questions [looks tired, “baseline” vs. “0H”: χ^2^(1) = 50.49, *p* < 0.001, “baseline” vs. “1H”: χ^2^(1) = 66.65, *p* < 0.001, “baseline” vs. “3H”: χ^2^(1) = 66.65, *p* < 0.001; looks old, “baseline” vs. “0H”: χ^2^(1) = 47.32, *p* < 0.001, “baseline” vs. “1H”: χ^2^(1) = 85.05, *p* < 0.001, “baseline” vs. “3H”: χ^2^(1) = 95.09, *p* < 0.001; looks irritated, “baseline” vs. “0H”: χ^2^(1) = 80.92, *p* < 0.001, “baseline” vs. “1H”: χ^2^(1) = 137.29, *p* < 0.001, “baseline” vs. “3H”: χ^2^(1) = 140.85, *p* < 0.001]. Next, comparing the selection rates of “baseline” and after stress load (“0H,” “1H,” and “3H”) in positive impression questions (“looks clean-cut” and “looks healthy”), the selection rate of “baseline” was significantly higher than “0H,” “1H,” and “3H” in both impression questions [looks clean-cut, “baseline” vs. “0H”: χ^2^(1) = 82.29, *p* < 0.001, “baseline” vs. “1H”: χ^2^(1) = 116.85, *p* < 0.001, “baseline” vs. “3H”: χ^2^(1) = 146.29, *p* < 0.001; looks healthy, “baseline” vs. “0H”: χ^2^(1) = 34.77, *p* < 0.001, “baseline” vs. “1H”: χ^2^(1) = 56.01, *p* < 0.001, “baseline” vs. “3H”: χ^2^(1) = 42.26, *p* < 0.001]. Conversely, there was no significant difference in the selection rate between “baseline” and “0H” in “looks good at work,” one of the positive questions. However, the selection rate of “baseline” was significantly higher than that of “1H” and “3H” [looks good at work, “baseline” vs. “1H”: χ^2^(1) = 4.17, *p* = 0.041, “baseline” vs. “3H”: χ^2^(1) = 7.00, *p* = 0.008].

## 4. Discussion

This study aimed to investigate the effect of short-term mild stress load on facial impression. First, a psychological stress load test was conducted to obtain facial images before and after stress loading. We compared heart rate and blood flow under the stress and rest conditions to confirm whether psychological stress load was applied. The heart rate during the Stroop test was significantly higher than at rest in the psychological stress load test (Figure 5). Heart rate, often used as an indicator of stress response in the autonomic nervous system, increases when the activity of the sympathetic nervous system increases (Skoluda et al., 2015). Hence, it was confirmed by the Stroop test that the sympathetic nervous system was in a dominant state; that is, psychological stress was experienced effectively. Similar to increased heart rate, peripheral vasoconstriction occurs when stress load increases sympathetic nerve activity (Chen and Lyga, 2014). Therefore, we confirmed whether the psychological stress load affected facial blood flow. As shown in Figure 6, cheek blood flow under the stress condition was significantly lower than that under the rest condition 1 hour after stress. Thus, short-term mild psychological stress caused not only an increase in heart rate but also a decrease in facial blood flow. It was shown that facial images acquired under the stress condition included the effects of stress load.

As mentioned above, facial images, including the effects of stress load, were obtained. Therefore, the effect of stress load on facial impressions was investigated using an online impression survey. Increasing the sample size can offset statistical errors caused by noise, such as room lighting and monitor settings (Hirao et al., 2021). In this survey, we compared facial impressions online by using 700 participants. An average computer-generated (CG) face was created from the participant’s facial photographs before stress loading (“baseline”) and after stress loading (“0H,” “1H,” and “3H”) and used for the survey. We hypothesized that stress causes negative impression changes such as “tired,” “old,” and “irritated.” We also set up the same number of positive impression questions to check for inconsistencies in participants’ responses. In terms of impression items such as “looks tired,” “looks old,” and “looks irritated,” the selection rate of the face image after stress load (“0H,” “1H,” and “3H”) was higher than that before stress load (“baseline”). Thus, psychological stress increases negative facial impressions. In addition, the selection rate of the face image before stress load (“baseline”) was significantly high for the impression items of “looks clean-cut” and “looks healthy.” In other words, positive facial impressions decreased owing to the stress load. These results were consistent with the results of negative questions. From the above, it was found that psychological stress load worsened facial impressions. Moreover, the deterioration of the impression was also confirmed in the face image after the stress load (“0H”), and the effect was maintained until the “3H” face image. In other words, changes in facial impressions caused by short-term mild psychological stress load may persist for several hours.

The created facial images included the effects of stress, reflected by an increased heart rate and decreased blood flow. However, it is possible that not only the psychological stress load but also the waiting time affected the continuation of the impression decline. In this study, participants waited in a seated position in the experimental room. The waiting time in the experimental room was at least 4 h for measurements from baseline to 3 h post-stress. This waiting time may have added additional psychological stress to the participants. Therefore, it is not clear how much the Stroop test and the waiting time individually contributed to the decrease in facial impression that lasted for 3 hours. We believe that conducting a survey using facial images acquired under the rest condition is one way to clarify the impact of waiting time on facial impressions. In other words, since the rest condition did not include a psychological stress load task, we can acquire facial images that reflect the stress caused by the waiting time. By conducting an impression survey using facial images obtained under the rest condition and comparing them with the results obtained under the stress condition, it will be possible to clarify the effect of waiting time on the duration of the decline in facial impressions. In this study, we did not conduct an impression survey using facial images in the rest condition, but further investigation is necessary.

One of the causes of the change in facial impression is considered to be a change in facial color due to a decrease in facial blood flow. There are blood vessels directly under the skin, and changes in facial blood flow may increase redness or, conversely, paleness. The blood flow decreased, and the facial skin color changed due to calculation tasks (Watanabe and Kageyama, 2017). Simultaneously, there are many reports on the relationship between skin color and impression. Increased redness in male faces has been shown to increase attractiveness through “healthiness” (Thorstenson et al., 2017). Notably, besides attractiveness, “aggression” and “dominance” are also affected by redness (Stephen et al., 2012). Furthermore, there was a positive correlation between yellowness in female faces and health (Henderson et al., 2016). The impression depended on the color of each part of the face and not the entire face (Jones et al., 2016). Therefore, there is a deep relationship between skin color and facial impression. In this study, stress load decreased facial blood flow. This may be the reason for the change in facial color. However, a significant decrease in facial blood flow occurred in “1H,” which was approximately 1 hour after the end of the stress load. However, the change in facial impression has been confirmed in the “0H” and is not linked to a significant decrease in blood flow. There are several hypotheses for this phenomenon, such as a slight change in blood flow causes a change in facial color, or the human eye perceives subtle changes in facial color, which affects impressions. Although the difference between the face images acquired was slight, a significant difference was observed in the selection rate in the face images before and after the stress load. Our results may indicate that humans are perceptually sensitive to facial differences and changes. It has been highlighted any skin condition changes, including skin color, can cause negative reactions and emotions in others (Orion and Wolf, 2014). It is believed that facial skin conditions have a large impact on impressions. It has been reported that humans are sensitive to changes in skin color and topography of the face, and may be able to detect slight changes in skin color (Matts et al., 2007; Samson et al., 2010b; Fink et al., 2012). Participants in this study may also have detected subtle differences in facial skin color before and after stress. In addition, even if the participants were not able to consciously discriminate differences in facial images, it is possible that the stimuli affected their impressions subconsciously because of subliminal perception. A previous study on subliminal perception of the face found that the color of the face unconsciously affects face recognition (Nakajima et al., 2015). Studies examining the effects of face images presented below the visibility threshold also revealed that adults subconsciously process the reliability of faces (Todorov et al., 2009). In this way, subliminal facial stimuli are known to affect facial recognition and impressions, and it is possible that facial recognition and impressions change even if a stimulus remains unconscious to humans. In order to clarify the cause of the impression change in this study, it is important to investigate whether the participants consciously recognized the difference in the image or whether they subconsciously evaluated the difference in impression. If subliminal perception is involved, clarifying its influence on face recognition and impression changes will help us to understand how subtle facial changes due to stress lead to impression changes. Additionally, other factors besides changes in skin color may be involved, such as changes in facial shape and expression. Further investigation is required on the factors that cause changes in facial impressions.

This study revealed that short-term mild psychological stress worsens facial impressions. Moreover, the effect persisted for several hours after the stress load. As mentioned above, it is possible that not only the psychological stress load but also the stress caused by waiting time affected the persistence of changes in facial impressions. Further investigation is needed to clarify the involvement of waiting time in the persistence of facial impression changes. In addition, the impression change occurred even though the difference between the face images was slight. Investigating the involvement of subliminal perception in face recognition and impressions will help us understand why slight differences in facial images before and after stress load cause changes in impressions. Further research is needed to clarify the causes of impression changes induced by psychological stress, including the role of subliminal perception. In addition, we used only male volunteers for face image creation before and after stress load to avoid the menstrual cycle’s influence. Therefore, similar results cannot be applied to female faces. Further research is needed to clarify the effects of stress on female facial impressions.

## 5. Conclusion

This study investigated the effects of short-term mild psychological stress on facial impressions. A psychological stress load test was conducted to acquire facial images that included the effects of stress. In the psychological stress load test, we confirmed an increase in heart rate and a decrease in blood flow, which are physiological responses to stress. From the impression survey results using facial images before and after stress loading, the impressions of facial images after stress loading worsened in almost all impression questions. Therefore, it was clarified that short-term mild psychological stress loading deteriorated facial impressions. From this study, we were able to obtain knowledge about the effects of everyday stress loads on facial impressions. We believe that this finding will be useful for the development of skincare and makeup products to improve the impression of individuals in a stressful society.

## Data availability statement

The datasets presented in this article are not readily available because confidentiality agreements with the participants; the data in this study are available only at the Shiseido Global Innovation Center. Requests to access the datasets should be directed to KK, koyo.koizumi@shiseido.com.

## Ethics statement

The studies involving human participants were reviewed and approved by the Research Ethics Committee of the Shiseido Global Innovation Center. The patients/participants provided their written informed consent to participate in this study.

## Author contributions

KK, NH, HY, and HO contributed to the conceptual design of the study. KK conducted the statistical analysis, and wrote the manuscript’s first draft with the support of NH. HO checked and revised the manuscript as the senior author. All authors contributed to the article and approved the submitted version.

## Funding

The present work was funded entirely by the Shiseido Brand Value R&D Institute and the Shiseido MIRAI Technology Institute.

## Conflict of interest

Authors KK, NH, and HY were employed by Shiseido Co., Ltd. The authors declare that this study received funding from Shiseido Brand Value R&D Institute and Shiseido MIRAI Technology Institute, Shiseido Co., Ltd. The funders had the following involvement in the study: The funders were involved in the study design, collection, analysis, interpretation of data, the writing of this article and the decision to submit it for publication

The remaining author declares that the research was conducted in the absence of any commercial or financial relationships that could be construed as a potential conflict of interest.

## Publisher’s note

All claims expressed in this article are solely those of the authors and do not necessarily represent those of their affiliated organizations, or those of the publisher, the editors and the reviewers. Any product that may be evaluated in this article, or claim that may be made by its manufacturer, is not guaranteed or endorsed by the publisher.
